# The neural basis of flashback formation: the impact of viewing trauma

**DOI:** 10.1017/S0033291712002358

**Published:** 2012-10-18

**Authors:** C. Bourne, C. E. Mackay, E. A. Holmes

**Affiliations:** 1 Department of Psychiatry, University of Oxford, Warneford Hospital, Oxford, UK; 2 FMRIB Centre, University of Oxford, Oxford, UK; 3 MRC Cognition and Brain Sciences Unit, Cambridge, UK

**Keywords:** Experimental psychopathology, flashbacks, functional magnetic resonance imaging, involuntary memory, memory, post-traumatic stress disorder, trauma

## Abstract

**Background:**

Psychological traumatic events, such as war or road traffic accidents, are widespread. A small but significant proportion of survivors develop post-traumatic stress disorder (PTSD). Distressing, sensory-based involuntary memories of trauma (henceforth ‘flashbacks’) are the hallmark symptom of PTSD. Understanding the development of flashbacks may aid their prevention. This work is the first to combine the trauma film paradigm (as an experimental analogue for flashback development) with neuroimaging to investigate the neural basis of flashback aetiology. We investigated the hypothesis that involuntary recall of trauma (flashback) is determined during the original event encoding.

**Method:**

A total of 22 healthy volunteers viewed a traumatic film whilst undergoing functional magnetic resonance imaging (fMRI). They kept a 1-week diary to record flashbacks to specific film scenes. Using a novel prospective fMRI design, we compared brain activation for those film scenes that subsequently induced flashbacks with both non-traumatic control scenes and scenes with traumatic content that did not elicit flashbacks (‘potentials’).

**Results:**

Encoding of scenes that later caused flashbacks was associated with widespread increases in activation, including in the amygdala, striatum, rostral anterior cingulate cortex, thalamus and ventral occipital cortex. The left inferior frontal gyrus and bilateral middle temporal gyrus also exhibited increased activation but only relative to ‘potentials’. Thus, these latter regions appeared to distinguish between traumatic content that subsequently flashed back and comparable content that did not.

**Conclusions:**

Results provide the first prospective evidence that the brain behaves differently whilst experiencing emotional events that will subsequently become involuntary memories – flashbacks. Understanding the neural basis of analogue flashback memory formation may aid the development of treatment interventions for this PTSD feature.

## Introduction

Post-traumatic stress disorder (PTSD) is the only psychiatric disorder for which a specific event and experience (the trauma and concurrent reaction) form part of the diagnosis (APA, 1994). Traumatic events include war, terrorist attacks, interpersonal violence and natural disasters (WHO, 2003). A small but clinically significant proportion of people who experience a trauma subsequently develop PTSD (Kessler *et al.* 1995). The hallmark PTSD symptom is the re-experiencing of the trauma in the form of intrusive memories (APA, 1994).

Intrusive, involuntary memories range from fleeting sensory impressions of traumatic events, to (very rarely) full-blown flashbacks which are so intense the patient dissociates and feels as if they are back at the time of the trauma. The former, and much more common, intrusive memories are clinically important in the maintenance of PTSD (Ehlers & Clark, 2000; Foa *et al.* 2007). Here we use the term ‘flashbacks’ to describe vivid, sensory–perceptual (predominantly visual images) emotional memories from a traumatic event that intrude involuntarily into consciousness. That is, a ‘flashback’ is ‘a mental vision of a past experience’ (Stein *et al.* 2007, p. 140).

Not all memories of trauma become flashbacks (Grey & Holmes, 2008). Flashback memories tend to be of distinct moments within an event, rather than of the whole trauma from start to finish. For example, a soldier may regularly experience traumatic scenes, such as of dead bodies or firefights. But these memories, despite being highly distressing, may never involuntarily intrude. However, the moment when he witnessed a comrade's arm being blown-off may repeatedly flash back. A key question is why some moments but not others become flashbacks? A neuroscientific approach raises the possibility that there may be differences in brain processing at the very time of experiencing the original trauma that predict which events within the trauma will subsequently become flashbacks.

The importance of psychological processing at the time of trauma (peri-traumatic) is emphasized by the diagnostic criteria for PTSD (APA, 1994), clinical psychology theories (Conway & Pleydell-Pearce, 2000; Ehlers & Clark, 2000; Brewin & Holmes, 2003) and meta-analysis of PTSD predictors (Ozer *et al.* 2003). However, neuroscientific data on the peri-traumatic phase are lacking. Studies using PTSD patients can only inform on post-traumatic processes, i.e. after flashbacks have already been established. Thus, there is no direct data to predict which brain regions may be involved in flashback aetiology, though patient studies may be informative.

A neurocircuitry model of PTSD (Rauch *et al.* 1998, 2006) highlights robust findings of reduced activity in ventromedial prefrontal regions, combined with increased activity in limbic regions. Shin *et al.* (2004) used positron emission tomography with 17 Vietnam veterans with PTSD, and 19 without. Script-driven imagery (playing back a patient's narrative of a neutral or traumatic event) was used to provoke PTSD symptoms. Compared with veterans without PTSD, those with the disorder had reduced activity in the medial prefrontal cortex and increased activity in the amygdala. Using a similar methodology, Rauch *et al.* (1996) reported increased blood flow for traumatic scripts compared with neutral scripts in limbic and paralimbic areas, including the amygdala. These findings have been replicated in functional magnetic resonance imaging (fMRI), positron emission tomography (PET) and single photon emission computed tomography (SPECT) studies (Hughes & Shin, 2011). Data relating to hippocampal function in PTSD are less clear, with some studies showing decreased function but others increased function (Francati *et al.* 2007). However, different processes may be involved peri-traumatically and post-traumatically. Thus, to understand flashback aetiology, we need to examine processing during trauma encoding.

It is clearly difficult to study people during real trauma for ethical and practical reasons. To this end, the trauma film paradigm is a well-established method used as an analogue model of psychological trauma (Horowitz, 1969; Holmes *et al.* 2004; Holmes & Bourne, 2008). Healthy individuals watch a film depicting traumatic events such as actual or threatened death or serious injury. Afterwards, a diary is used to record involuntary flashbacks of segments of the film. Healthy individuals often experience several flashbacks during the following week. However, as with real-life trauma, individual responses to the same trauma film are highly idiosyncratic. One person may report flashbacks to a scene where a fireman carried a baby, whereas another may flash back to a man's face covered in blood. A critical question remains: what is happening peri-traumatically at a neural level while witnessing scenes that later intrude involuntarily?

**Fig. 1.**
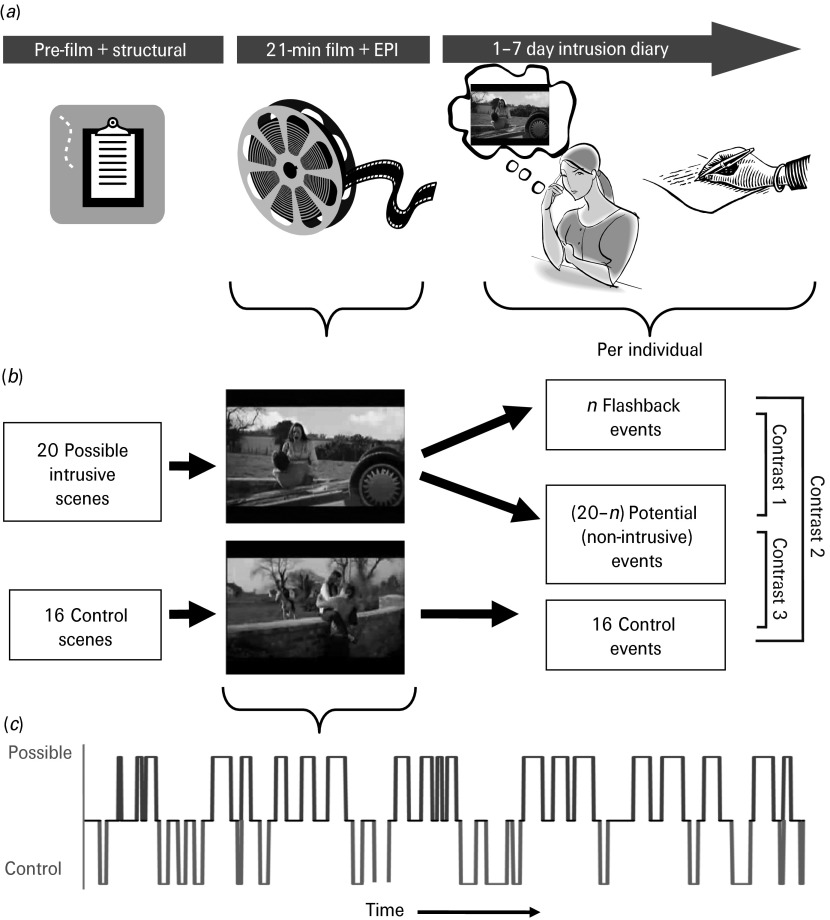
Study design. (*a*) The three stages of the study: pre-film; film viewing (peri-traumatic encoding) and echo planar imaging (EPI); and the 1-week diary (flashback recording). (*b*) Modelling of the three functional magnetic resonance imaging (fMRI) event types. For each participant, an idiosyncratic subset ‘*n*’ of the 20 possible intrusive scenes was experienced as analogue flashbacks in the diary and become flashback events. This establishes ‘20 – *n*’, the remainder of the possible scenes that did not flash back as potential events. None of the 16 control scenes flashed back for any individual. Thus, for each participant three event types were modelled: (1) flashback intrusive events; (2) potential events; and (3) control events. (*c*) fMRI task schematic: Frequency, timing and duration of the 20 possible intrusive scenes (which later become either flashback or potential events) and the 16 control scenes within the film timeline.

This study is the first to integrate the trauma film paradigm with a unique prospective fMRI design (Fig. 1). Brain scanning was performed whilst volunteers watched a traumatic film, then for the next week a diary was used to determine, for each individual, those film moments that later became flashbacks. Thus, fMRI events are only defined 1 week after scanning on the basis of the later cognitive experience. Using this design we provide the first test of what happens at the time an emotional flashback is formed (i.e. at encoding). Moreover, the paradigm addresses a little-understood feature of human memory more generally – why do some moments spring to mind as if out of the blue (involuntarily) while others do not? This is important since though there is vast literature on emotional memory, very little is known about the subset of emotional memories that intrude involuntarily.

A whole-brain approach was taken with exploratory hypotheses. Based on both patient memory recall studies as well as non-clinical studies about the encoding and consolidation of deliberate (voluntary) memories (e.g. Wagner *et al.* 1998; Canli *et al.* 2000; Kensinger & Corkin, 2004; McGaugh, 2004; Francati *et al.* 2007; Roozendaal *et al.* 2008; Hughes & Shin, 2011), we predicted that encoding of traumatic material would involve the amygdala, hippocampus, and prefrontal and temporal cortices. In particular, we used a unique, prospective fMRI design to investigate the hypothesis that involuntary recall of trauma (flashback) is determined during the original encoding of the event. In support of this method, we note that prospective fMRI has been employed elsewhere. For example, studies that consider the ‘subsequent memory effect’ (e.g. Wagner *et al.* 1998; Kensinger & Corkin, 2004) also differentiate brain activity at one time point on the basis of subsequent behavioural events (see also Ramel *et al.* 2007). The unique aspects here relate to the use of video stimuli, the use of highly emotional and distressing content, the focus on intrusive, involuntary memory encoding and the combination of predefined stimuli types (possible flashback scenes *versus* control scenes) with subsequent behaviourally defined stimuli types (i.e. actual flashback scenes *versus* those possible scenes that did not flash back).

## Method

### Participants

A total of 22 healthy volunteers (mean age = 22 years, s.d. = 3.08; 17 female, five male) were recruited from the local community. Given the stressful environment of the MRI scanner and the film content, vulnerable individuals were excluded by pre-screening using the Beck Depression Inventory-II (Beck *et al.* 1996) and the trait scale of the Spielberger State-Trait Anxiety Inventory (STAI; Spielberger *et al.* 1983). All participants declared no current or previous psychiatric illness (including PTSD) or substance misuse. Due to ethical considerations, all recruitment material provided information about the nature of the film, specifically that it contained scenes of a traumatic or potentially distressing nature, e.g. the aftermath of real-life road traffic accidents and surgical procedures. The study was approved by the NHS Oxfordshire Research Ethics Committee ‘B’ and participants provided their written informed consent.

### Questionnaire measures

Participants completed pre-film state measures: the state scale of the STAI (Spielberger *et al.* 1983); the 19 subject-rated items from the Clinician Administered Dissociative States Scale (Bremner *et al.* 1998); and visual analogue scales for four emotions: horrified, sad, calm and happy. These measures were repeated post-film (see Supplementary material: Table S1 and Impact of Trauma Film).

### Trauma film viewing

The film lasted for 21 min and was presented using Presentation v12.01 software (www.neurobs.com) (Fig. 1). The trauma film was adapted for fMRI from previous behavioural experiments (Holmes *et al.* 2004, 2009; Stuart *et al.* 2006; Bourne *et al.* 2010). It comprised 15 different short video clips and was constructed so that 20 possible intrusive scenes (scene length: 5–37 s, mean = 22.5 s) and 16 control scenes (scene length: 5–36 s, mean = 16.4 s) were distributed fairly evenly throughout without disrupting the narrative of the individual videos with at least 6 s between any possible or control scene. Scene length was matched as closely as possible between control and possible scenes and did not significantly differ (*t*_34_ = 1.94, n.s.). Scene order was arranged to ensure that no more than four possible scenes or four control scenes occurred in a row before at least one of the alternative scene type occurred (see Fig. 1*c*).

Scene type was defined using a database of diary-based intrusion data (matched to film content) from several hundred participants who had previously watched the sections of the film (Holmes *et al.* 2004, 2009; Stuart *et al.* 2006; Bourne *et al.* 2010). Scenes were selected as possible intrusive scenes if they had been found to produce intrusions in at least some people in previous studies or control if no one had ever previously experienced an intrusion of that scene. In summary, all possible scenes included traumatic material (e.g. a fireman carrying a baby's body from a car wreckage) and would later, on the basis of the diary, be divided into those that did ‘flash back’ *versus* ‘potentials’ that did not (providing a non-intrusive trauma comparison). Pre-defined control scenes were selected if they did not contain traumatic content either from scenes from the same video clip (e.g. other people walking near the same car wreckage, without a visible death or injury) or from different footage that matched as much as possible the themes of the potentially intrusive sequences (i.e. close-up of a woman applying eye make-up was matched to a close-up of eye surgery).

### Flashback diary for 1 week

Participants used a diary to record any intrusive flashback memories of the film during the 7 days following the experimental session (Holmes *et al.* 2004, 2009; Stuart *et al.* 2006; Bourne *et al.* 2010). To ensure that the intrusions were an analogue of PTSD flashback memories, participants were advised (both verbally and via written instructions in the diary) that intrusions have sensory properties (mental images, i.e. not just verbal thoughts) and are those that come to mind unbidden (i.e. not those deliberately recalled). Further, participants were asked to write down each intrusion's content (e.g. ‘fireman carrying a baby’; ‘dead body after being trampled by elephant’; ‘the small circular imprints being pulled on the eye’) in as much detail as possible. These flashback descriptions were later individually checked to ensure that they matched in content to the content of the trauma film (rather than another film or past experience) and to aid localization of the specific time point in the film for that intrusion for the subsequent fMRI analysis.

Participants returned for a follow-up session 1 week later. They rated their overall compliance with completing the diary in terms of ‘incompleteness’ and ‘accuracy’ on 100 mm visual analogue scales anchored from ‘not at all’ to ‘extremely’ (Holmes *et al.* 2004). Next, each intrusion in the diary was discussed to confirm that it met the criteria for flashback memories (spontaneous, image-based, of the film) and was then matched to a specific film sequence. For example, the flashback of a fireman carrying a baby was matched to the one scene in the film where this event happened. It is noted that since the film comprised distinct scenes (e.g. a fireman carrying a baby; a rampaging elephant; an eye operation) rather than continuous footage of just one event this facilitated the identification of which scene a flashback matched to or not (and discrimination from possible autobiographical life experiences).

This matching process determined which of the possible scenes had become flashback scenes for each participant. Two independent judges performed this matching process and agreed on 76 out of 77 identified separate intrusive events in the diaries, an agreement rate of 98.7%. An inter-rater reliability analysis using the κ statistic (Landis & Koch, 1977; Fleiss, 1981) was performed (κ = 0.94, *p* < 0.001), suggesting an excellent level of reliability for matching of diary entries to film scenes and that the flashbacks reported were indeed of the film (rather than other events).

For fMRI analysis, the diaries provided three main explanatory variables for each participant (see Fig. 1): (i) flashback events (i.e. those film sequences that were recorded in the diary as having intruded spontaneously for the participant); (ii) potential events (i.e. those film sequences identified from previous studies as being possibly intrusive but that were not experienced as flashback intrusive events for that participant); and (iii) control events (i.e. the 16 scenes identified as unlikely to provoke intrusions). Return of the diary data confirmed that none of the control events was experienced as flashbacks for any participant. The primary contrast of interest was ‘flashback’ *versus* ‘potential’ scenes, as this distinguishes encoding a flashback from viewing non-intrusive traumatic material. Differences in this contrast would show whether the brain responds differently to traumatic emotional events that later flash back. Additionally, both flashback and potential scenes were compared with control scenes.

### Heart rate and emotional content covariates

To control for potential confounding effects of differential heart rate response to, or subjective emotional experience of, the flashback and potential scenes, heart rate was measured throughout the film and fMRI scan and participants provided subjective emotionality ratings of each of the 20 possible scenes at follow-up. These measures were used as covariates in the analysis (see Supplementary material: Covarying for Heart Rate Response and Covarying for Emotional Content). Covariate data for one participant were not available.

### Image acquisition

Echo planar imaging data were acquired on a 3-T Siemens TIM Trio System with a 12-channel head coil [45 slices, voxel resolution = 3×3 × 3 mm^3^, repetition time (TR) = 3 s, echo time (TE) = 30 ms, flip angle = 87°]. T1-weighted structural images were acquired for subject alignment using a magnetization prepared rapid gradient echo (MPRAGE) sequence (voxel resolution 1 × 1×1 mm^3^, TR = 2040 ms, TE = 4.7 ms).

### Image analysis

fMRI analysis was carried out using FEAT (FMRI Expert Analysis Tool) version 5.91 [part of the Functional Magnetic Resonance Imaging of the Brain (FMRIB)'s Software Library, www.fmrib.ox.ac.uk/fsl]. Data were pre-processed using FEAT default options: motion correction was applied using rigid body registration to the central volume; Gaussian spatial smoothing was applied with a full width half maximum of 5 mm; brain matter was segmented from non-brain using a mesh deformation approach; high-pass temporal filtering was applied with a cut-off of 100 s. For each individual, the three main event types were specified in the general linear model: flashback, potential and control scenes, with the duration of each individual scene also modelled. These events were modelled by convolving with a double-γ haemodynamic response function. A fourth variable representing a nuisance regressor was used to remove time periods when participants were reading instructions. The analysis was performed both with and without the inclusion of heart rate data and emotionality ratings for each of the 20 possible scenes as alternative fifth regressors. Additionally, the temporal derivates of these regressors were included. All regressors were included in a general linear model applied voxel-wise to the pre-processed, pre-whitened imaging data. Time-series statistical analysis was carried out using FILM (FMRIB's Improved. Linear Model) with local autocorrelation correction (Woolrich *et al.* 2001). The appropriate contrasts of interest were constructed from the four basic regressors and were registered to the Montreal Neurological Institute (MNI) 152 template using affine transformation. Voxel-wise group analysis was carried out in MNI standard space using FLAME (FMRIB's Local Analysis of Mixed Effects) stage 1 (Beckmann *et al.* 2003; Woolrich *et al.* 2004; Woolrich, 2008). The resulting *z* statistic images were thresholded using *z* > 2.3 and a family-wise error-corrected cluster significance threshold of *p* < 0.05 (Forman *et al.* 1995) was applied.

### Region of interest (ROI) analysis

*Post hoc* ROI analysis was performed to understand better the large number of brain regions that demonstrated significant increases in activation in response to flashback *versus* potential scenes. We extracted ROIs for the amygdala, accumbens, putamen, rostral anterior cingulate cortex (rACC), thalamus, ventral occipital cortex and the hippocampus due to our hypotheses. Except for the left inferior frontal gyrus (left IFG) and middle temporal gyrus (MTG), the ROIs were extracted on an anatomical basis using the Harvard–Oxford Cortical or Sub-cortical atlas and were specified as being within the 20–100% probability maps for the relevant structure. For the left IFG and MTG, the ROIs were extracted by masking those areas that were significantly activated on a whole-brain basis in the flashback *versus* potential contrast but were not within the flashback *versus* control contrast. All ROIs (except for the hippocampus) contained clusters of significant activation for the flashback *versus* potential contrast on a whole-brain basis and *post hoc* analysis examined the nature of the differences relative to the control condition. As such, a repeated-measures analysis of variance was conducted for each structure thresholded at *p* < 0.05.

## Results

### Flashbacks in the 1-week diary

The trauma film successfully induced flashbacks in all participants. The mean total flashback frequency over 1 week was 6.82 (s.d. = 9.96). Some scenes were re-experienced more than once during the week. Thus, the mean number of different events modelled in the fMRI analysis (flashbacks) per participant was 3.50 (s.d. = 2.63, range = 1–9; see Supplementary material: Fig. S1a), leaving an average of 16.5 potential scenes per participant. Of the 20 possible scenes, 16 became flashbacks for at least one of the participants and no single possible scene became a flashback for more than half of the participants. Diary compliance scores suggest that participants maintained their intrusion diaries diligently and none was excluded for poor compliance (incompleteness ratings: mean = 0.63, s.d. = 0.60; accuracy: mean = 8.30, s.d. = 0.88).

### Whole-brain analysis

**Fig. 2.**
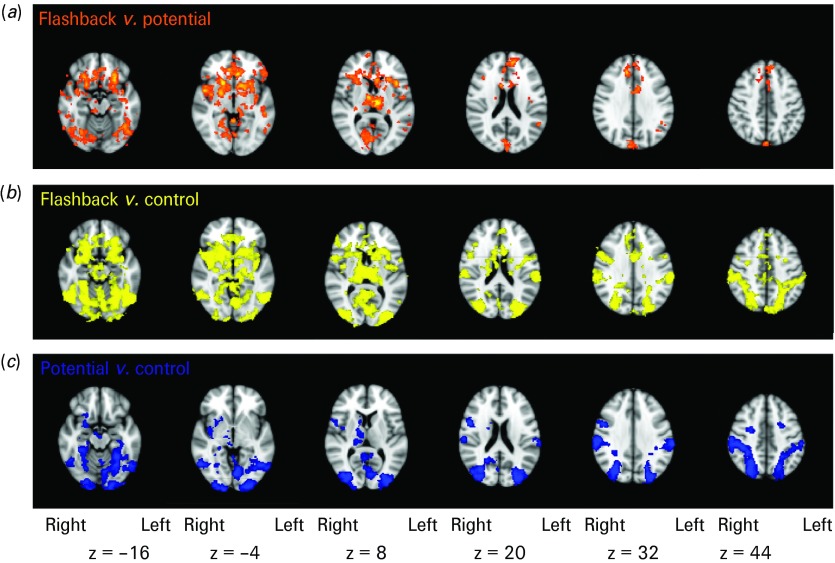
Contrast analysis of flashback *versus* potential *versus* control film scenes. Contrast analysis showing areas of increased blood oxygen level-dependent (BOLD) response (*p* < 0.05 cluster corrected) rendered onto the Montreal Neurological Institute (MNI) 2 mm standard brain. (*a*) Areas with greater activation when viewing flashback relative to potential scenes (orange). (*b*) Areas with greater activation when viewing flashback relative to control scenes (yellow). (*c*) Areas with greater activation when viewing potential relative to control scenes (blue).

When viewing flashback scenes relative to potential scenes (Fig. 2; top row), increased activation was found in widespread areas of the brain including the amygdala, thalamus, striatum, rACC and ventral occipital cortex. Additionally, there was increased activation in the left IFG and bilaterally in the MTG. There were no areas of activation for the reverse contrast.

When viewing flashback scenes relative to control scenes (Fig. 2; middle row), increased activation was found in the amygdala, thalamus, striatum, rACC and ventral occipital cortex. However, there was no increased activation in the left IFG and MTG. There were no areas of activation for the reverse contrast.

Finally, the areas of increased activation when viewing potential relative to control scenes (Fig. 2; bottom row) were similar to the flashback *versus* control contrast (middle row) with respect to posterior brain areas, but lacked the frontal activations present in the flashback *versus* control contrast (see Supplementary material: Fig. S2). Again, there were no areas of activation for the reverse contrast.

Peak voxel coordinates for these activations are shown in the Supplementary material (Tables S2, S3 and S4). Coronal views of these contrasts are also shown in the Supplementary material (Fig. S2).

Analysis was also performed to control for potential confounding effects of physiological and subjective emotional responses. The same pattern of results was found with both of the covariates (with slightly reduced spatial extent) as was initially found. Therefore these variables are unlikely to explain the activation pattern associated with flashback encoding.

### ROI analysis

**Fig. 3.**
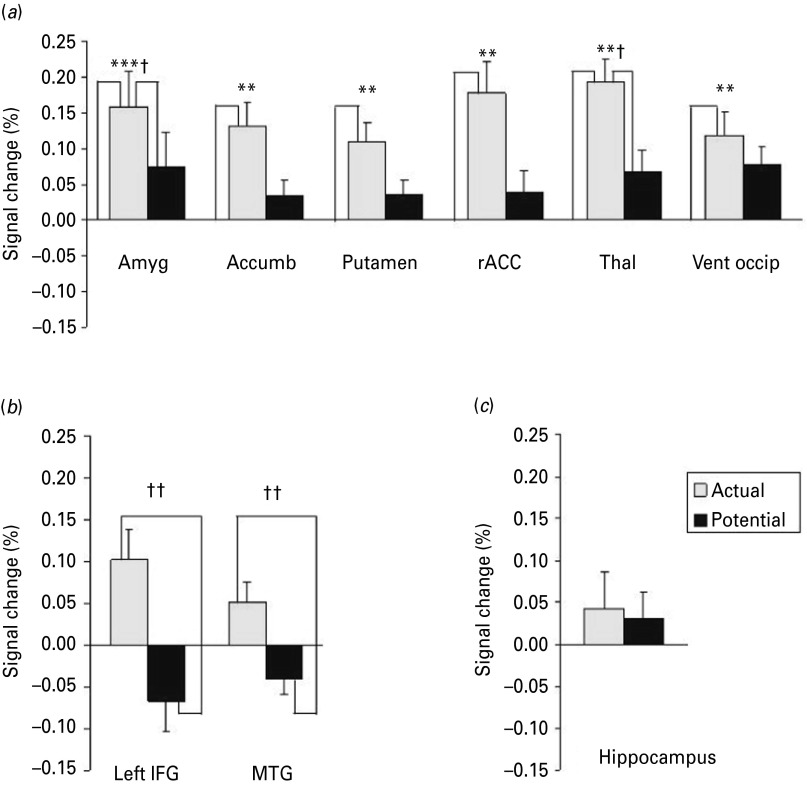
Pattern of blood oxygen level-dependent (BOLD) signal change for flashback and potential relative to control events in key brain regions. (*a*) ‘Dose effect’ pattern of signal change in the amygdala (Amyg), accumbens (Accumb), putamen, rostral anterior cingulate cortex (rACC), thalamus (thal) and ventral occipital cortex (Vent occip). (*b*) Differential effect in the left inferior frontal gyrus (IFG) and middle temporal gyrus (MTG). (*c*) No effect in the hippocampus. Values are means, with standard errors represented by vertical bars. Mean value was significantly different from that for the control scenes: ** *p* < 0.01, *** *p* < 0.001 (*p* values Bonferroni-corrected). Mean value was significantly different from that for the potential scenes: † *p* < 0.05, †† *p* < 0.01 (*p* values Bonferroni-corrected).

Fig. 2 shows that while viewing traumatic information, many brain regions responded more to flashback scenes than to potential scenes. To understand further the blood oxygen level-dependent (BOLD) response in these brain areas, the mean percentage BOLD signal change across each ROI was plotted for the flashback and potential scenes relative to the control scenes (Fig. 3).

**Fig. 4.**
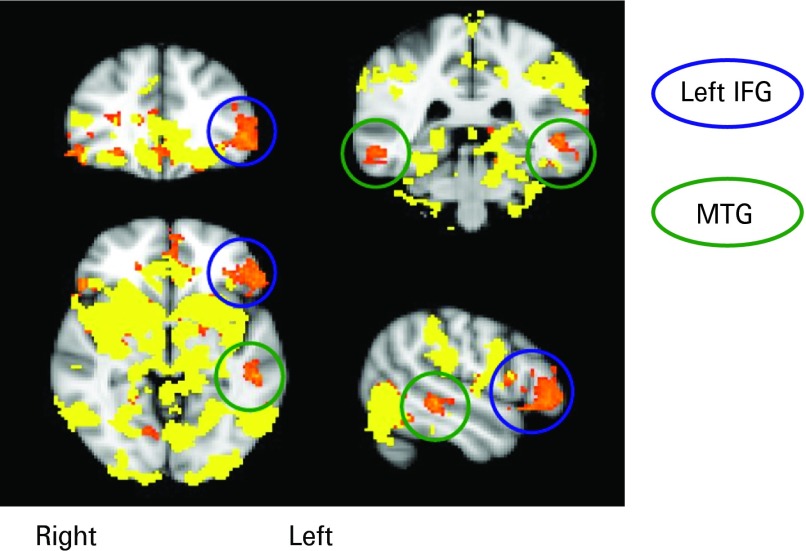
Overlay of flashback *versus* control contrast (yellow) on flashback *versus* potential contrast (orange). This highlights the left inferior frontal gyrus (IFG) and middle temporal gyrus (MTG). These areas appear only in the flashback *versus* potential contrast.

The amygdala and thalamus responded to flashback scenes with increased levels of activation relative to both potential scenes (amygdala: *t*_21_ = 3.51, *p* = 0.018); thalamus: *t*_21_ = 3.43, *p* = 0.027) and control scenes (amygdala: *t*_21_ = 4.99, *p* = 0.0006; thalamus: *t*_21_ = 5.97, *p* = 0.0042) (see Fig. 3*a*). Several other brain regions showed an increased BOLD response during flashback scenes relative to control scenes (accumbens: *t*_21_ = 4.14, *p* = 0.0042; putamen: *t*_21_ = 4.39, *p* = 0.0023; rACC: *t*_21_ = 4.04, *p* = 0.009; and ventral occipital: *t*_21_ = 3.75, *p* = 0.009) but not relative to potential scenes (accumbens: *t*_21_ = 2.66, *p* = 0.13; putamen: *t*_21_ = 2.71, *p* = 0.12; rACC: *t*_21_ = 2.64, *p* = 0.14) although these regions showed significant differences for the latter contrast on a whole-brain basis. Additionally, two further brain regions, the left IFG and bilateral MTG (Fig. 3*b*), also exhibited increased levels of activation for flashback scenes relative to potential scenes (left IFG: *t*_21_ = 3.79, *p* = 0.009; MTG: *t*_21_ = 3.78, *p* = 0.009). As in the whole-brain analysis (see Fig. 4), these two regions did not show an increased BOLD signal for flashback scenes relative to control scenes (left IFG: *t*_21_ = 2.87, *p* = 0.081; MTG: *t*_21_ = 2.21, *p* = 0.34) or for potential relative to control scenes (left IFG: *t*_21_ = 1.90, *p* = 0.65; MTG: *t*_21_ = 2.09, *p* = 0.44). As can be seen in Fig. 3*c*, there was no significant difference between activation in the hippocampus for either flashback (*t*_21_ = 1.01, *p* = 0.72) or potential (*t*_21_ = 0.85, *p* = 0.40) scenes relative to control scenes, or for flashback relative to potential scenes (*t*_21_ = 0.36, *p* = 0.72). All *t* tests were two-tailed and Bonferroni corrected for multiple testing.

## Discussion

This study is the first to our knowledge to examine the neural activity at the time of an analogue trauma experience that gives rise to subsequent involuntary memories – flashbacks. A better understanding of peri-traumatic processing (i.e. at the time of trauma) is important given the uniqueness of PTSD compared with all other psychiatric diagnoses in requiring a specific event to have occurred for the diagnosis to be given. Although this study could only investigate the neural processing of an experimental analogue of a traumatic event, studies such as this may provide a useful insight into peri-traumatic processing given the ethical and practical difficulties of conducting a similar study during real trauma. The prospective design involved brain scanning during a traumatic film, then using a diary to determine for each individual those film moments which later became flashbacks over the subsequent week. Flashback scenes were compared with similar events that did not flash back (potential) and control scenes. The key finding was that brain activation at the time of encoding a film segment that later flashed back was significantly increased across widespread brain regions including the amygdala, ventral occipital cortex, striatum, rACC, thalamus, left IFG, and MTG. Far from being specific, the data suggest the importance of peri-traumatic processing in widespread brain regions in determining which moments within a trauma will later flash back.

Many brain regions exhibited elevated activation during flashback encoding (relative to both potential and control scenes) including: those associated with emotional processing (amygdala) (Phelps *et al.* 2004), higher-level visual processing and mental imagery (ventral occipital cortex) (Kosslyn *et al.* 2001), and threat (rACC) (Bishop *et al.* 2004). This is consistent with the nature of flashbacks as highly emotional, visual memories of threatening situations. Since there are no prospective studies to date that have investigated trauma encoding and the aetiology of flashback in actual PTSD patients, any parallels drawn to the existing literature should be made with caution. Nevertheless, PTSD patient symptom provocation studies have consistently implicated the amygdala (Bremner *et al.* 1999; Shin *et al.* 2006; Koenigs *et al.* 2007; Yehuda & LeDoux, 2007) and less consistently, the hippocampus (Francati *et al.* 2007). The role of the amygdala and dorsal medial PFC/dorsal anterior cingulate cortex in anxiety and fear is well documented (e.g. McGaugh, 2004; Mechias *et al.* 2010; Robinson *et al.* 2012). In particular, studies have implicated hyperresponsivity in these regions as a risk factor for PTSD (Shin *et al.* 2011) and abnormal functioning in these areas as a mechanism explaining impaired fear extinction in PTSD (Milad *et al.* 2009). Therefore, it is of interest that these regions show increased activation for flashback and potential encoding relative to control scenes. However, these regions also showed greater activation for flashback *versus* potential scenes, perhaps implying a role for these regions specifically in involuntary encoding.

Interestingly, activation levels within the left IFG and MTG appeared to distinguish between traumatic content that subsequently flashed back and similar content that did not. The interpretation of these findings warrants further consideration, in particular to explain why these areas tend to exhibit lower activation for the ‘potential’ (highly emotionally charged) stimuli compared with the control ones. An imaging finding often raised in clinical teaching on PTSD is that early PET studies found that when shown pictures of combat, Vietnam veterans had decreased regional cerebral blood flow (rCBF) in Broca's area (Shin *et al.* 1997, 1999). Decreased rCBF has also been found in the anterior portion of Broca's area (BA 45/46) in PTSD patients who visualized their trauma (Rauch *et al.* 1996). Speculatively, this has some similarity to our region in the left IFG. Additionally, increased activation at encoding in left prefrontal areas (including the left IFG) and temporal areas (including the MTG) predicts later deliberate recall in ‘subsequent memory effect’ studies for both non-emotional and emotional pictorial stimuli (Wagner *et al.* 1998; Kensinger & Corkin, 2004). These brain regions are thought to flag ‘to be remembered’ events. In evolutionary terms, enhanced memory for life-threatening events would clearly be useful. It remains to be established whether such an effect for voluntary recall also applies to involuntary memories. Our data suggest this possibility.

There are several methodological considerations for this study. First, other factors may have contributed to the activations identified. For example, emotionality is by definition part of a traumatic stimulus (APA, 1994). We covaried for emotionality as well as heart rate (which is part of an arousal reaction but could affect the BOLD response) in all analyses and neither variable altered the pattern of results. The activations with and without heart rate were very similar and provide reassurance that the activation differences are not merely due to corresponding changes in vasculature. Thus, although intense emotional reaction may be a necessary condition for flashback formation it appears not to be sufficient.

Second, while the mean number of flashback events induced was consistent with analogue (Holmes *et al.* 2004; Bourne *et al.* 2010) and clinical findings (Grey & Holmes, 2008), it is low compared with numbers normally required in fMRI event-based designs. Conventional wisdom holds that at least 10 events would be required to power an fMRI paradigm sufficiently (Jezzard *et al.* 2001); however, here, between one and nine were modelled (mean = 3.5). Although one might predict that this number would be insufficient for statistical power, we found a surprisingly large BOLD response on a per event basis, sufficient to produce the extent and magnitude of activation evident in Fig. 2 (*z*_21_ = 2.3–4.4 for flashback *versus* potential; and *z*_21_ = 2.3–4.8 for flashback *versus* control, all statistically significant when fully corrected for multiple comparisons). Although the number of events does not affect the parameter estimate in a general linear model (i.e. separate regressors for different trial types), having fewer events will increase variance in the modelled response and therefore limit statistical power. Indeed, a significant linear relationship was found between the number of events and signal variance for each participant for the flashback *versus* potential contrast (*F*_1,20_ = 10.23, *p* = 0.006, *R*^2^ = 0.34) (see Supplementary material: Fig. S1b). To ensure that the results were not confounded by errors due to the very small number of events in some individuals, we conducted subanalyses of those subjects who had a greater number of flashback events. The areas of significant difference survived the reduction in power caused by reducing the number of subjects (see Supplementary material: Sub-analyses of Whole-Brain Data by Number of Flashback Events and Fig. S1). Thus, the activation pattern associated with flashback events was robust, and this bodes well for using tailored fMRI to investigate other clinical phenomena. It is also noted that in the current study the diary compliance ratings were high and, further, that the two independent judges who matched each flashback to a specific scene in the film showed high levels of agreement. Inaccuracies or omissions in the diary data, or poor levels of flashback match to film would add noise to the fMRI data and work against finding any meaningful results.

A third consideration relates to the validity of an analogue trauma as a model for flashback development. The content of the film met DSM-IV criteria (APA, 1994) for traumatic events. In addition to directly experiencing, we note that witnessing death and serious injury is consistent with criterion A1 for PTSD (APA, 1994). Further, indirect exposure to trauma such as television footage of the 9/11 terrorist attacks has been associated with (albeit low rates of) post-traumatic symptoms (Breslau *et al.* 2010). An intense emotional response is criterion A2 for PTSD (APA, 1994) and the film induced significant increases in state anxiety, state dissociation and horror (see Supplementary material: Table S1), though clearly less so than during real trauma. The recording of flashbacks in a diary is also an analogue of clinical practice, where patients can be asked to record their flashbacks each week for later discussion in a cognitive behavioural therapy (CBT) session. Overall, the real-time medium of emotional film appears to have good face validity as a trauma analogue when compared with experimental alternatives such as static pictures or fear conditioning to abstract shapes.

Emotional involuntary memories of trauma constitute just one of the clinical symptoms of PTSD (APA, 1994) (others include avoidance and hyperarousal). However, better understanding of flashback formation has implications for our understanding of the aetiology of PTSD, even if only as part of the puzzle. Indeed the status of flashbacks themselves is the subject of great interest, and better understanding their genesis may relate to our understanding of how culture-bound they may be (Jones *et al.* 2003). Interestingly, our findings may also have relevance for our basic understanding of human involuntary memory (Mace, 2007); we know only a little about involuntary memory recall (Hall *et al.* 2008) and even less about its encoding. A cognitive science perspective suggests that intrusive sensory memories of emotional events may indeed be considered as occurring on a continuum with PTSD flashbacks at an extreme (Berntsen, 2001; Rubin *et al.* 2008).

Understanding aetiology of the hallmark symptom of PTSD may help contribute to preventative treatment development. Notably, there are currently no evidence-based preventative treatments in the immediate aftermath of trauma, that is the first few hours or early days after a trauma, which is concerning given the scale of trauma worldwide. This has been highlighted in recent reviews of both psychological (Agorastos *et al.* 2011) and pharmacological (Bisson, 2007) treatments. The National Institute for Clinical Excellence (2005) guideline on PTSD recommends ‘watchful waiting’ for the first 4 weeks post-trauma. Roberts *et al.* (2009) conclude that further research is required to evaluate the most effective ways of providing psychological help in the early stages after a traumatic event. In contrast, there are well-developed treatments for when PTSD is diagnosed after 1 month, such as trauma-focused CBT (e.g. Ehlers & Clark, 2000). Roberts *et al.* (2010) suggest that trauma-focused CBT may be useful to treat acute stress reactions within 3 months post-trauma. Furthermore, trauma-focused CBT may even be preventative of chronic reactions (Kornør *et al.* 2008). However, earlier-stage preventative interventions are still required for the first few hours or early days after trauma.

The current neural findings underscore the importance of peri-traumatic encoding for later flashbacks. Memory is labile for several hours after encoding (Nader *et al.* 2000), thus it may be possible to impede flashback memory consolidation in the immediate aftermath of trauma (Ressler & Mayberg, 2007) by targeting the associated neural circuitry. This could be done using pharmacological means targeting the noradrenergic system to make an impact on emotional processing and the amygdala (Pitman *et al.* 2002), or by using cognitive methods such as disrupting visual processing (Holmes *et al.* 2009) or emotional regulation (Ehlers *et al.* 2003).

In conclusion, to our knowledge, this is the first study to attempt to examine the neurological processes involved in the encoding of both traumatic material in general and involuntary memories in particular. We predicted that neural activity at the time of experiencing a traumatic event is important for later memory flashbacks. Our data show that particular brain responses at the time of viewing traumatic material are associated with those moments that subsequently intrude days later. Such memory intrusions or flashbacks are at the core of PTSD. Understanding the mechanisms of involuntary memories and flashbacks is of critical importance in developing our models of PTSD. An understanding of flashback memory formation will aid development of an experimental medicine model for this PTSD symptom, and inform its possible prevention. Since Proust's quintessential recall of ‘the Madeleine’, the images of scenes conjured up in emotional involuntary memories have featured as part of a psychological landscape we need to better understand. Thus, this new paradigm helps sheds light on a fundamental aspect of human memory – why particular emotional sensory memories may spring to mind when we do not want them to.

## Supplementary Material

Supplementary Material Supplementary information supplied by authors.

## Supplementary material

For supplementary material accompanying this paper visit http://dx.doi.org/10.1017/S0033291712002358.
