# Supplementary material for: The neural basis of flashback formation: the impact of viewing trauma
Source: Psychol Med. 2012 Oct 18;43(7):1521–32. doi: 10.1017/S0033291712002358 (PMC3806039; doi:10.1017/S0033291712002358)
Supplement: Supplementary Material — Supplementary information supplied by authors. [file S0033291712002358sup001.doc]

**SUPPLEMENTARY INFORMATION**

*Impact of Trauma Film.*

Viewing the film induced a mood deterioration as predicted. Mood change was assessed by state mood measures administered immediately before and after viewing the film in the scanner:

*State Anxiety.* State scale of the STAI (Spielberger *et al.* 1983). Participants completed this 20-item scale based upon how they felt “right now”.

*Mood Measures.* Visual analogue scales (VAS) were used to assess 4 different emotions: horrified, sad, calm, and happy. Each emotion was rated from 0 “*Not at all*” to 10 “*Extremely*” for feelings “right now”.

*State Dissociation.* The 19 subject-rated items from the Clinician Administered Dissociative States Scale (Bremner e*t al.* 1998) were rated on a 5-point scale ranging from 0 “*Not at all*” to 4 “*Extremely”* for feelings “right now”.

The mean (standard deviation) pre-film and post-film scores on these measures are shown in Table S1.

From pre-film to post-film there was an increase in state anxiety [*t*(21) = 3.26, *p* = .004, *d* = 0.75], state dissociation [*t*(21) = 3.54, *p* = .002, *d* = 0.66], sadness [*t*(21) = 4.35, *p* < .001, *d* = 0.98], and horror [*t*(21) = 5.06, *p* < .001, *d* = 1.50], and a decrease in calm [*t*(21) = 2.89, *p* = .009, *d* = 0.67] and happiness [*t*(21) = 3.52, *p* = .002, *d* = 0.90].

Table S1. Means and standard deviations for state mood measures pre and post-film

|  | *Pre-film* | *Post-film* | *Paired Samples t-test of means* |
| --- | --- | --- | --- |
| State Measures | M SD | M SD | t(21) p |
| State Anxiety (20-80) | 31.22 7.14 | 39.00 12.86 | 3.26 .004 |
| State Dissociation (0-76) | 4.05 5.17 | 9.41 10.18 | 3.54 .002 |
| VAS Horrified (0-10) | 0.14 0.28 | 2.65 2.36 | 5.06 < .001 |
| VAS Sad (0-10) | 1.08 1.14 | 3.04 2.59 | 4.35 < .001 |
| VAS Calm (0-10) | 6.89 1.47 | 5.41 2.77 | 2.89 .009 |
| VAS Happy (0-10) | 6.16 1.43 | 4.59 2.02 | 3.52 .002 |

*Covarying for Heart Rate Response.*

In case there was a different physiological response to Flashback, Potential, and Control scenes which drove the BOLD response in our contrast analysis, each participant’s heart rate was measured throughout the film and the fMRI data acquisition. Heart rate data was not available for one participant and so this individual was excluded from the covariate analysis. Heart rate was measured by a Siemens MR compatible pulse oximeter fitted to the participant’s right index finger and converted into a R-wave via a MR Equipment Corporation Multigas Monitor 9500. This data was then passed through a Cambridge Electronics Design Analogue to Digital Converter (Micro 1401) and the output recorded in Spike 2 software ([www.ced.co.uk/pru.shtml](http://www.ced.co.uk/pru.shtml)) sampling at 50Hz. The Spike 2 output file was converted into a rolling average heart rate in beats per minute every second (averaged across 6 seconds). This raw data was inspected for noise and signal drop out with any data points below 40 bpm or above 120bpm being replaced by the average of the proceeding and subsequent values. Each participant’s heart rate data was de-meaned and used to weight each second of the fMRI data as an additional event in the GLM model. The pattern of results for each of the 3 contrasts (Flashback v Potential; Flashback v Control; and Potential v Control) with this additional heart rate covariate was very similar to the original analysis without this extra regressor.

*Covarying for Emotional Content.*

Although some Potential scenes were not intrusive for a specific individual, they had previously been shown to be intrusive for some individuals (Holmes *et al.* 2004; Stuart *et al.* 2006; Holmes *et al.* 2009) or were for other individuals in the current study. Therefore, Potential scenes were more similar in emotionality to an individual’s Flashback events than the Control film scenes and effectively served as a control for emotionality. However, there was still the possibility that what made specific scenes become intrusive was the presence of more distressing or emotional content as experienced by a specific individual. Therefore, at the follow-up session, participants were given short written descriptions for each of the 20 Possible intrusive scenes and asked to rate how distressed and emotional they found each of them. (These emotionality ratings were not available for one participant and so this individual was excluded from the covariate analysis). Items were rated on a scale from 0 “*Not at all*” to 10 “*Extremely*”.

We acknowledge that this measure was retrospective and may have been contaminated by the presence of intrusive recollections for the Flashback events in the intervening week. However, any contamination effect would likely have increased the retrospective rating of the emotionality of intruding scenes thereby reducing the contrast effect once emotionality was co-varied. In any event, it was not practical to conduct the emotional rating test immediately after film viewing as such post-film tasks would be predicted to disrupt artificially the natural memory consolidation process and alter number of flashbacks in the intrusion diary (Flashback events; Krans *et al.* 2009). Once each individual’s emotionality ratings were obtained, the ratings were de-meaned and used to weight each of the 20 Possible scenes as an additional event in the GLM model. The pattern of results for each of the 3 contrasts (Flashback v Potential; Flashback v Control; and Potential v Control) with this additional emotionality covariate was very similar to the original analysis without this extra regressor.

*Sub-analyses of Whole Brain Data by Number of Flashback Events.*

Figure S1a shows the frequency of Flashback events across the participants. Figure S1b shows the relationship between the number of Flashbacks and variability in the estimation of the contrast. Due to concerns over the relatively low mean number of Flashback events giving rise to a false positive result a number of sub-set analyses were conducted. Figure S1c shows the results of this sub-analyses for a single representative slice (z = -4). Each column in Figure S2 represents a different sub-set of participants. The sub-set with the highest mean number of Flashback events (mean = 6.1) is shown on the far right for those participants with at least 4 Flashback events (N=9). To the left is the next highest sub set (mean = 5.5), participants with at least 3 Flashbacks (N=11) and to the left of this column participants with at least 2 Flashbacks (N=16; mean = 4.4). The left hand column is the original analysis of the full data-set. The three rows correspond to the three whole brain contrasts shown in Figure 2.

Whilst the exact spatial extent of the activations patterns varies between sub-sets, the overall pattern is remarkably similar across all the columns especially for the key contrast of Flashback v Potential. This suggests that the signal detection is robust and not due to false negatives arising from a low mean number of Flashback events in the original analysis of the whole data-set.

**
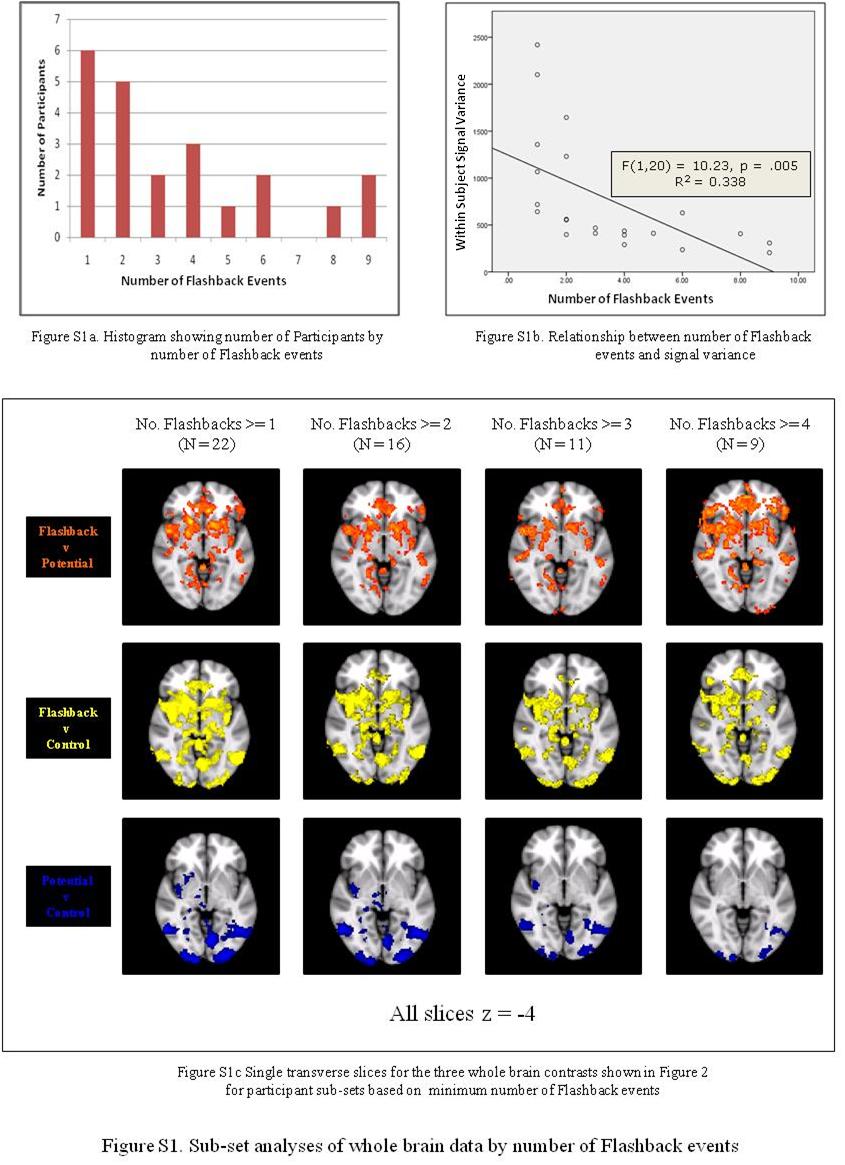
**

Activation peak coordinates for whole brain analysis.

| Table S2 | Activation peak coordinates for areas significantly more activated for Actual Intrusive Events versus Potential Events | | | | | | | | | | | |
| --- | --- | --- | --- | --- | --- | --- | --- | --- | --- | --- | --- | --- |
| **Brain region** | | **Laterality** |  | **MNI coordinates** | | |  | **Cluster Size** | | **Z** | **P value** |  |
|  | |  |  | |  |  | | **(voxels)** | | **score** |  |  |
|  | |  | x | | y | z | |  |  | |  |  |
|  | |  |  | |  |  | |  |  | |  |  |
| Thalamus | | L | -4 | | -14 | 6 | | 17525 | 4.47 | | < .0001 |  |
|  | |  |  | |  |  | |  |  | |  |  |
| Putamen | | R | 18 | | 16 | -4 | |  | 4.16 | |  |  |
|  | |  |  | |  |  | |  |  | |  |  |
| Lingual gyrus | | R | 8 | | -68 | 4 | |  | 4.04 | |  |  |
|  | |  |  | |  |  | |  |  | |  |  |
| Frontal Pole | | R | 44 | | 56 | 26 | |  | 3.98 | |  |  |
|  | |  |  | |  |  | |  |  | |  |  |
| Anterior cingulate (ACC) | | L | -6 | | 32 | -2 | |  | 3.93 | |  |  |
|  | |  |  | |  |  | |  |  | |  |  |
| Inferior temporal gyrus | | L | -42 | | -56 | -12 | | 1070 | 4.09 | | < .0001 |  |
|  | |  |  | |  |  | |  |  | |  |  |
| Temporal occipital cortex | | L | -34 | | -62 | -21 | |  | 3.65 | |  |  |
|  | |  |  | |  |  | |  |  | |  |  |
| Occipital fusiform gyrus | | L | -30 | | -66 | -20 | |  | 3.5 | |  |  |
|  | |  |  | |  |  | |  |  | |  |  |
| Posterior supramarginal gyrus | | L | -56 | | -50 | 26 | | 389 | 3.25 | | 0.024 |  |
|  | |  |  | |  |  | |  |  | |  |  |
| Angular gyrus | | L | -52 | | -54 | 20 | |  | 3.1 | |  |  |
|  | |  |  | |  |  | |  |  | |  |  |
| Lateral occipital cortex | | L | -50 | | -64 | 26 | |  | 2.97 | |  |  |

L, left; R, right

| Table S3 | Activation peak coordinates for areas significantly more activated for Actual Intrusive Events versus Control Events | | | | | | | | | | | |
| --- | --- | --- | --- | --- | --- | --- | --- | --- | --- | --- | --- | --- |
| **Brain region** | | **Laterality** |  | **MNI coordinates** | | |  | **Cluster Size** | | **Z** | **P value** |  |
|  | |  |  | |  |  | | **(voxels)** | | **score** |  |  |
|  | |  | x | | y | z | |  |  | |  |  |
|  | |  |  | |  |  | |  |  | |  |  |
| Lateral inferior occipital cortex | | L | -52 | | -66 | -6 | | 58217 | 4.85 | | < .0001 |  |
|  | |  |  | |  |  | |  |  | |  |  |
| Lateral superior occipital cortex | | L | -32 | | -84 | 22 | |  | 4.84 | |  |  |
|  | |  |  | |  |  | |  |  | |  |  |
| Insular | | R | 38 | | -4 | -8 | |  | 4.79 | |  |  |
|  | |  |  | |  |  | |  |  | |  |  |
| Occipital fusiform gyrus | | L | -22 | | -68 | -12 | |  | 4.76 | |  |  |
|  | |  |  | |  |  | |  |  | |  |  |
| Thalamus | | L | -4 | | -16 | 4 | |  | 4.75 | |  |  |
|  | |  |  | |  |  | |  |  | |  |  |
| Frontal Pole | | L | -42 | | -56 | -12 | |  | 4.72 | |  |  |

L, left; R, right

| Table S4 | Activation peak coordinates for areas significantly more activated for Potential Intrusive Events versus Control Events | | | | | | | | | | | |
| --- | --- | --- | --- | --- | --- | --- | --- | --- | --- | --- | --- | --- |
| **Brain region** | | **Laterality** |  | **MNI coordinates** | | |  | **Cluster Size** | | **Z** | **P value** |  |
|  | |  |  | |  |  | | **(voxels)** | | **score** |  |  |
|  | |  | x | | y | z | |  |  | |  |  |
| Lateral superior occipital cortex | | R | 32 | | -72 | 30 | | 27026 | 5.28 | | < .00001 |  |
|  | |  |  | |  |  | |  |  | |  |  |
| Lingual gyrus | | L | -4 | | -76 | -2 | |  | 4.96 | |  |  |
|  | |  |  | |  |  | |  |  | |  |  |
| Superior parietal lobule | | R | 24 | | -56 | 62 | |  | 4.95 | |  |  |
|  | |  |  | |  |  | |  |  | |  |  |
| Inferior frontal gyrus (IFG) | | R | 50 | | 6 | 20 | | 1485 | 4.46 | | < .00001 |  |
|  | |  |  | |  |  | |  |  | |  |  |
| Precentral gyrus | | R | 48 | | 4 | 30 | |  | 4.16 | |  |  |
|  | |  |  | |  |  | |  |  | |  |  |
| Insular | | R | 40 | | -6 | -8 | |  | 3.92 | |  |  |
|  | |  |  | |  |  | |  |  | |  |  |
| Putamen | | R | 20 | | 14 | -10 | |  | 3.23 | |  |  |
|  | |  |  | |  |  | |  |  | |  |  |
| Precentral gyrus | | R | 28 | | -8 | 48 | | 1024 | 4.43 | | < .00001 |  |
|  | |  |  | |  |  | |  |  | |  |  |
| Superior frontal gyrus | | R | 28 | | 8 | 62 | |  | 3.32 | |  |  |
|  | |  |  | |  |  | |  |  | |  |  |
| Middle frontal gyrus | | R | 28 | | 14 | 48 | |  | 3.13 | |  |  |
|  | |  |  | |  |  | |  |  | |  |  |
| Thalamus | | R | 14 | | -8 | 8 | | 641 | 3.29 | | 0.0016 |  |
|  | |  |  | |  |  | |  |  | |  |  |
| Caudate | | R | 12 | | 8 | 10 | |  | 3.17 | |  |  |
|  | |  |  | |  |  | |  |  | |  |  |

L, left; R, right

*S6. Additional images for whole brain analysis*

*
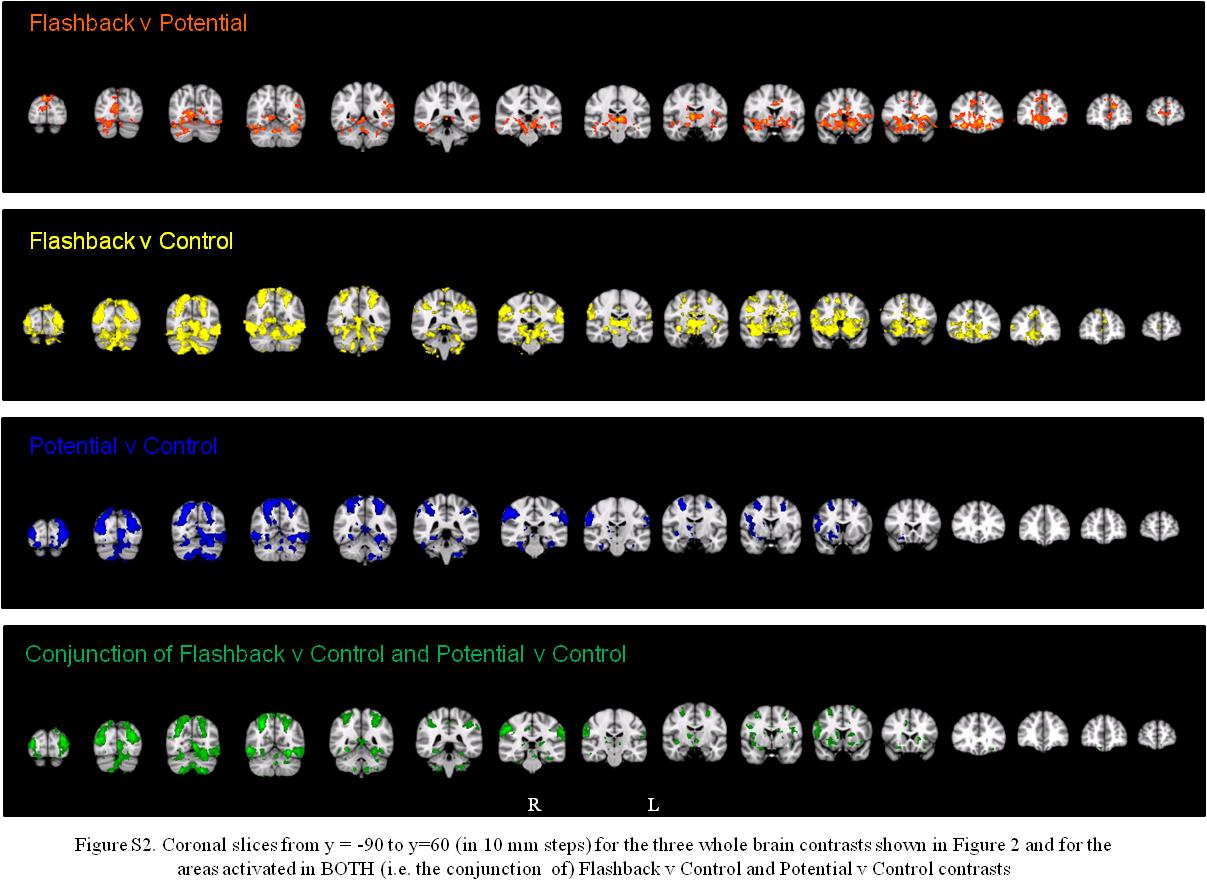
*

References

**Bremner JD, Krystal JH, Putnam FW, Southwick SM, Marmar CR, Charney DS, Mazure CM** (1998). Measurement of dissociative states with the clinician-administered dissociative states scales (CADSS). *Journal of Traumatic Stress* **11**, 125-136.

**Holmes EA, Brewin CR, Hennessy RG** (2004). Trauma films, information processing, and intrusive memory development. *Journal of Experimental Psychology: General* **133**, 3-22.

**Holmes EA, James EL, Coode-Bate T, Deeprose C** (2009). Can Playing the Computer Game 'Tetris' Reduce the Build-up of Flashbacks for Trauma? A Proposal from Cognitive Science. *PLoS ONE* **4**, e4153 doi:10.1371/journal.pone.0004153.

**Krans J, Naring G, Holmes EA, Becker ES** (2009). Tell me more: can a memory test reduce analogue traumatic intrusions? *Behaviour Research and Therapy* **47**, 426-430.

**Spielberger CD, Gorsuch RL, Lushene R, Vagg, PR, Jacobs, GA** (1983). *Manual for State-Trait Anxiety Inventory*. Consulting Psychologists Press: Palo Alto, CA.

**Stuart ADP, Holmes EA, Brewin CR** (2006). The influence of a visuospatial grounding task on intrusive images of a traumatic film. *Behaviour Research and Therapy* **44**, 611-619.
